# Glycemic control with empagliflozin, a novel selective SGLT2 inhibitor, ameliorates cardiovascular injury and cognitive dysfunction in obese and type 2 diabetic mice

**DOI:** 10.1186/s12933-014-0148-1

**Published:** 2014-10-26

**Authors:** Bowen Lin, Nobutaka Koibuchi, Yu Hasegawa, Daisuke Sueta, Kensuke Toyama, Ken Uekawa, MingJie Ma, Takashi Nakagawa, Hiroaki Kusaka, Shokei Kim-Mitsuyama

**Affiliations:** Department of Pharmacology and Molecular Therapeutics, Kumamoto University Graduate School of Medical Sciences, 1-1-1 Honjyo, Kumamoto, 860-8556 Japan

**Keywords:** Cardiovascular complications, Vascular dysfunction, Cognitive decline, Oxidative stress, Inflammation

## Abstract

**Background:**

There has been uncertainty regarding the benefit of glycemic control with antidiabetic agents in prevention of diabetic macrovascular disease. Further development of novel antidiabetic agents is essential for overcoming the burden of diabetic macrovascular disease. The renal sodium glucose co-transporter 2 (SGLT2) inhibitor is a novel antihyperglycemic agent for treatment of type 2 diabetes. This work was performed to determine whether empagliflozin, a novel SGLT2 inhibitor, can ameliorate cardiovascular injury and cognitive decline in db/db mouse, a model of obesity and type 2 diabetes.

**Methods:**

*(1) Short-term experiment:* The first experiment was performed to examine the effect of 7 days of empagliflozin treatment on urinary glucose excretion and urinary electrolyte excretion in db/db mice. *(2) Long-term experiment:* The second experiment was undertaken to examine the effect of 10 weeks of empagliflozin treatment on cardiovascular injury, vascular dysfunction, cognitive decline, and renal injury in db/db mice.

**Results:**

*(1) Short-term experiment:* Empagliflozin administration significantly increased urinary glucose excretion, urine volume, and urinary sodium excretion in db/db mice on day 1, but did not increase these parameters from day 2. However, blood glucose levels in db/db mice were continuously decreased by empagliflozin throughout 7 days of the treatment. *(2) Long-term experiment:* Empagliflozin treatment caused sustained decrease in blood glucose in db/db mice throughout 10 weeks of the treatment and significantly slowed the progression of type 2 diabetes. Empagliflozin significantly ameliorated cardiac interstitial fibrosis, pericoronary arterial fibrosis, coronary arterial thickening, cardiac macrophage infiltration, and the impairment of vascular dilating function in db/db mice, and these beneficial effects of empagliflozin were associated with attenuation of oxidative stress in cardiovascular tissue of db/db mice. Furthermore, empagliflozin significantly prevented the impairment of cognitive function in db/db mice, which was associated with the attenuation of cerebral oxidative stress and the increase in cerebral brain-derived neurotrophic factor. Empagliflozin ameliorated albuminuria, and glomerular injury in db/db mice.

**Conclusions:**

Glycemic control with empagliflozin significantly ameliorated cardiovascular injury and remodeling, vascular dysfunction, and cognitive decline in obese and type 2 diabetic mice. Thus, empagliflozin seems to be potentially a promising therapeutic agent for diabetic macrovascular disease and cognitive decline.

## Background

Type 2 diabetes is a major risk factor for cardiovascular disease and cardiovascular disease is the leading cause of mortality in patients with diabetes [1,2]. Previous large clinical trials have established that strict glycemic control significantly reduces diabetic microvascular complications such as nephropathy or retinopathy [3-5]. On the other hand, to date there has been uncertainty regarding whether any current antihyperglycemic agents can actually reduce cardiovascular event, because most of previous clinical trials indicated no benefit of current antihyperglycemic agents in prevention of cardiovascular disease [3-8]. Moreover, diabetes is a major risk factor for cognitive decline as well as cardiovascular disease [9-11]. However, no information is available regarding the effect of antihyperglycemic agents on cognitive decline in diabetes. Hence, further development of other novel pharmacological approaches of glycemic control is necessary to reduce macrovascular disease and cognitive impairment in patients with diabetes.

The kidney plays a key role in glucose homeostasis and has recently become a target organ for treatment of diabetes. Under normal conditions, the kidney reabsorbs all the glucose from the glomerular filtrate and back into the blood. Sodium glucose co-transporter 2 (SGLT2) is located in the brush border membrane of the proximal convoluted tubule of the nephron, and mediates the majority of glucose reabsorption from glomerular filtrate [12,13]. Pharmacological inhibition of SGLT2 increases urinary glucose excretion (UGE) and consequently decreases blood glucose levels in an insulin-independent manner [12,13]. Thus, SGLT2 inhibitors represent a novel class of antihyperglycemic drugs and have recently become available for treatment of patients with type 2 diabetes [12-15]. It is a future clinical key issue whether SGLT2 inhibitor can prevent macrovascular complication, cognitive decline, or microvascular complication in diabetic patients. Previous preclinical studies using diabetic animal models support the notion that SGLT2 inhibitors may exert protective effects against diabetic nephropathy (diabetic microvascular disease) [16-21]. However, to the best of our knowledge, the effect of SGLT2 inhibition on cardiovascular disease and cognitive function in diabetes remains to be explored.

Empagliflozin [22-24] is a novel inhibitor of SGLT2, and is characterized by highly selective and potent inhibitor of SGLT2, compared with other SGLT2 inhibitors. In the present experimental study, we hypothesized that glycemic control with empagliflozin can ameliorate cardiovascular injury and cognitive decline in obese and type 2 diabetic mice. To demonstrate our hypothesis, we examined the effects of long-term empagliflozin treatment on cardiac fibrosis and inflammation, coronary arterial remodeling, vascular dysfunction, cardiovascular oxidative stress, and learning and reference/working memory in db/db mice, a useful model of obesity and type 2 diabetes. We obtained the evidence that empagliflozin may be a promising therapeutic agent for diabetic macrovascular disease and cognitive decline.

## Methods

### Animals

All procedures were performed in accordance with institutional guidelines for animal research and were approved by the Animal Care and Use Committee of Kumamoto University. Male db/db mice (C57BLKS/J-lepr^db^/lepr^db^) and male nondiabetic and lean db/m mice (C57BLKS/J-lepr^db^/+) as control were purchased from Japan Charles River Laboratories Japan Inc. (Yokohama, Japan). All animals were housed in an animal facility with a 12-hour light-dark cycle and were given the standard chow and water ad libitum.

### Drugs

Empagliflozin [22-24], a selective sodium glucose cotransporter-2 (SGLT2) inhibitor, was kindly gifted from Boehringer Ingelheim Pharma GmbH & Co.KG, Germany.

### Experiment I: Effect of short-term (7 days) empagliflozin administration

The main objective of Experiment I was to investigate the effect of short-term (7 days) empagliflozin treatment on urinary glucose excretion, urine volume, urinary electrolytes, and blood glucose. db/db mice at 7 weeks of age already displayed significant obesity and type 2 diabetes. Therefore, in this study, drug treatment of db/db mice was initiated from 7 weeks of age. db/db mice were acclimatized to the metabolic cages to collect 24-hour urine samples every day from 5 days before the experiment, randomly assigned 2 groups, and were given (1) the standard diet (MF diet, ORIENTAL YEAST Co., Ltd, Tokyo, Japan) (2) the standard diet containing 0.03% empagliflozin for 7 days and 24-hour urine samples were collected from each mouse every day. Non fasting blood glucose was measured before and 1, 3, and 7 days after start of empagliflozin administration.

Furthermore, the effect of short-term empagliflozin treatment was also examined in non-diabetic db/m mice in the same manner as the above mentioned experiment on db/db mice.

### Experiment II: Effect of long-term (10 weeks) empagliflozin administration on cardiovascular complications, cognitive function, and renal injury

**Figure 1:**
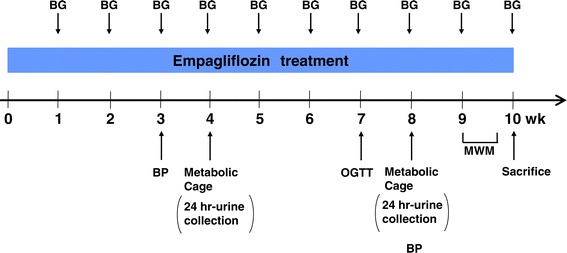
**The design of Experiment II.** Abbreviations used: BG, measurement of blood glucose; BP, measurement of blood pressure; OGTT, oral glucose tolerance test; MWM, Moris water maze test.

Experiment II was performed to mainly investigate the effects of long term empagliflozin treatment on cardiovascular injury, vascular dysfunction, cognitive dysfunction, and renal injury. Seven-week-old db/db mice were assigned to 2 groups, and were given (1) the standard diet (MF diet, ORIENTAL YEAST Co., Ltd, Tokyo, Japan) or (2) the standard diet containing 0.03% empagliflozin in the same manner as the Experiment I, and drug treatment was performed for 10 weeks. The design of the Experiment II is shown in Figure 1. Body weight and blood glucose were periodically measured every week throughout the treatment. Blood samples were collected from the tail vein of mice for measurement of non-fasting blood glucose. Blood pressure (BP) was measured at 3 and 8 weeks after the start of drug treatment. After 4 and 8 weeks of drug treatment, mice were housed in metabolic cages to collect 24-hour urine for measurement of urinary glucose, urinary electrolytes, urinary albumin, and urinary creatinine excretions. Oral glucose tolerance test was performed after 7 weeks of drug treatment. Morris water maze test (MWM) was performed at 9 weeks of drug treatment. At the end of 10 weeks of treatment, 17-week-old db/db mice were anaesthetized with isoflurane, the blood was collected by cardiac puncture, to measure serum insulin and glucose. The heart, thoracic aorta and kidney were rapidly excised to perform histological and biochemical examinations, as described below in detail.

### Experiment III: Effect of empagliflozin administration on cerebral oxidative stresss and brain-derived neurotrophic factor of db/db mice

To examine the effect of empagliflozin on cerebral oxidative stress and brain-derived neurotrophic factor (BDNF) in db/db mice, empagliflozin treatment was performed on db/db mice for 16 days in the same manner as the above experiment, and cerebral tissue was collected from each mouse by the modification of our previous method [25].

### Measurement of blood pressure

Blood pressure was measured by tail-cuff plethysmography (BP-98A; Softron Co, Tokyo, Japan).

### Oral glucose tolerance test

Mice were deprived of food for overnight and then orally given glucose (1 mg/g body weight). Tail vein bloods were taken from mice at 0, 30, 60 and 120 min after glucose administration to measure serum glucose concentrations. Blood glucose concentrations were measured by a portable glucose meter (Sanwa Kagaku Kenkyusho CO., LTD, Nagoya, Japan).

### Vessel ring preparation and organ chamber experiments

Isometric tension studies were performed, as previously described [26]. In brief, thoracic aortas from mice were cut into 5 mm rings, and mounted in organ baths filled with modified Tyrode buffer (pH 7.4; NaCl 121 mmol/l, KCl 5.9 mmol/l, CaCl_2_ 2.5 mmol/l, MgCl_2_ 1.2 mmol/l, NaH_2_PO_4_ 1.2 mmol/l, NaHCO_3_ 15.5 mmol/l, and D (+)-glucose 11.5 mmol/l) aerated with 95% O_2_ and 5% CO_2_ at 37^°^C. The preparations were attached to a force transducer, and isometric tension was recorded on a polygraph. A resting tension of 1 g was maintained throughout the experiment. Vessel rings were precontracted with L-phenylephrine (10^−7^ mol/l). After the plateau was attained, the rings were exposed to increasing concentrations of acetylcholine (10^−9^ mol/l to 10^−4^ mol/l) or S-nitroso-N-acetylpenicillamine (SNAP) (10^−9^ mol/l to 10^−4^ mol/l) to obtain cumulative concentration-response curves.

### Measurement of tissue superoxide

Heart, aorta, kidney, and brain removed from mice, were immediately frozen in Tissue-Tek O.C.T. embedding medium (Sakura Finetek, Tokyo, Japan). Dihydroethidium (DHE) was used to evaluate tissue superoxide levels in situ, as described [27]. DHE fluorescence of each tissue section was quantified by Lumina Vision version 2.2 (Mitani corporation, Tokyo, Japan). The mean fluorescence was quantified and expressed relative to values obtained from control mice.

### Measurement of cerebral 8-hydroxy-deoxyguanosine(8-OHdG)

For measurement of cerebral 8-OHdG, cerebral DNA was extracted with DNA Extractor TIS kit (Wako Pure Chemical Industries, Ltd. Osaka, Japan) and cerebral 8-OHdG was measured by New 8-OHdG Check ELISA (Japan Institute for the Control of Aging, NIKKEN SEIL CO., Ltd. Tokyo, Japan).

### Histological and immunohistochemical analysis

Hearts were fixed in 4% (wt/vol.) paraformaldehyde, embedded in paraffin, sectioned 5-μm slices, and stained with Sirius Red F3BA (0.5% wt/vol. in saturated aqueous picric acid; Aldrich Chemical Company, St Louis, MO, USA) for the measurement of cardiac interstitial fibrosis, pericoronary arterial fibrosis and coronary arterial thickness. The area of fibrosis and coronary arterial thickness was analyzed by Lumina Vision version 2.2 (Mitani corporation, Tokyo, Japan). Kidneys were fixed in 4% paraformaldehyde, embedded in paraffin, sectioned 5-μm slices, and stained with periodic acid-Schiff (PAS). Glomerular sclerosis was assessed in PAS stained kidney sections by a semiquantitative score (grades 0 to +4), as previously described [28]. Fifty glomeruli were randomly selected from each mouse to perform the analysis.

For detection of cardiac and glomerular macrophage infiltration, frozen cardiac and kidney sections were incubated overnight with the primary antibody(rat anti-mouse CD68, Serotec; ×1000) followed by anti-rat secondary antibody (BioSource, Camarillo, CA, USA), as described previously [28].

### Morris water maze test

Learning and reference/working memory were evaluated by the Morris water maze (MWM) test, as previously described [29]. Groups were blinded to the examiners. In brief, swimming paths were video-tracked with a camera fixed on the ceiling of the room and analyzed by the software (Muromachi Kikai, Tokyo, Japan). A training session was carried out before the hidden platform test sessions. Mice were given 60 seconds free swimming and guided to climb onto the hidden platform and allowed to remain there for 30 seconds before returning to their cages. On the hidden platform test, the mice had 5 sessions at 20-minute intervals per day on the following 4 consecutive days (day 1 to 4). During each session mice were released from randomly assigned 3 starting points and swam for 100 seconds. On the probe test at day 5, the hidden platform was removed and the mice swam freely for 100 seconds. The number of times the mice crossed the original platform location was recorded. On the visible platform test which was performed after the probe test on day 5, the platform was elevated 5 cm above the water surface level and placed in a different position. The mice were given four sessions of a visible trial with an inter-session interval of 20 minutes.

### Western blot analysis

Western blot analysis of cerebral tissue protein was performed by our previous method described [30]. Antibodies used were as follows: anti-gp91phox (91kDa) (x2000, Santa Cruz Biotechnology, Inc., Santa Cruz, CA, USA), anti-p67phox (67kDa) (x5000, BD Biosciences, San Jose, CA, USA), anti-BDNF (14kDa) (x2000, Santa Cruz Biotechnology, Inc., Santa Cruz, CA, USA), anti-GAPDH (37kDa) (x5000, Santa Cruz Biotechnology, Inc., Santa Cruz, CA, USA). The intensity of the bands was quantified using NIH Image analysis software v1.61. In individual samples, each value was corrected for that of GAPDH.

### Analysis of biochemistry

Serum insulin levels were quantified by using a commercial ELISA kit (Morinaga, Tokyo, Japan). Urine biochemistry were performed at SRL Inc (Tokyo, Japan).

### Statistical analysis

Statistical analysis was performed using GraphPad Prism version 5.02 for Windows (GraphPad Software Inc., San Diego, CA) and Ekuseru-Tokei 2012 statistical software (Social Survey Research Information Co, Ltd, Tokyo, Japan). Data were presented as mean ± SEM. The data on time course experiments were analyzed by two-way ANOVA with repeated measures followed by Bonferroni post hoc test for multiple comparisons. Statistical significance was determined with one-way ANOVA, followed by the Newman–Keuls post hoc test between each group. Data were analyzed with the Kruskal-Wallis test followed by Steel-Dwass post hoc test, when similar variances were not obtained among comparison groups or a normal distribution was not confirmed among comparison groups. In all tests, differences were considered statistically significant at a value of P < 0.05.

## Results

### The effects of short-term (7 days) empagliflozin treatment on blood glucose, urinary parameters and body weight of db/db mice and non-diabetic db/m mice

**Figure 2:**
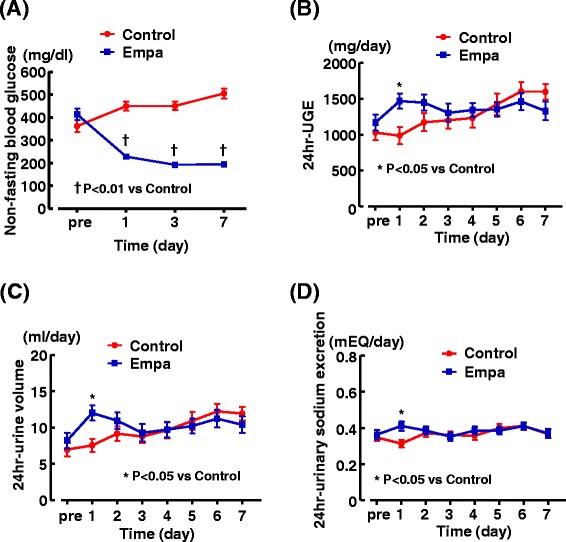
**Effects of short-term (7 days) empagliflozin administration on non-fasting blood glucose (A), 24 hr-urinary glucose excretion (B), 24 hr-urine volume (C) and 24 hr-urinary sodium excretion (D) in db/db mice.** Twenty four-hour urine of each mouse was collected with metabolic cages every day before and throughout the experiment. Abbreviations used: UGE, urinary glucose excretion; control, control (untreated) db/db mice; Empa, empagliflozin-treated db/db mice; pre, the data obtained before start of drug treatment. *p < 0.05, †p < 0.01 vs control db/db mice. Values are mean ± SEM (n = 10-11).

Figure 2 shows the time course of non-fasting blood glucose, 24-hr urinary glucose excretion, 24-hr urine volume and 24-hr urinary sodium excretion in control and empagliflozin-treated db/db mice. Non-fasting blood glucose of db/db mice was already significantly reduced on day 1 after start of empagliflozin administration and remained significantly decreased throughout 7 days of the treatment (Figure 2 (A)). Only on day 1, 24-hr urinary glucose excretion, urine volume and urinary sodium excretion (in Figure 2 (B), (C), and (D), respectively) were significantly increased in db/db mice given empagliflozin, but these parameters did not differ between empagliflozin and control groups from day 2.

**Figure 3:**
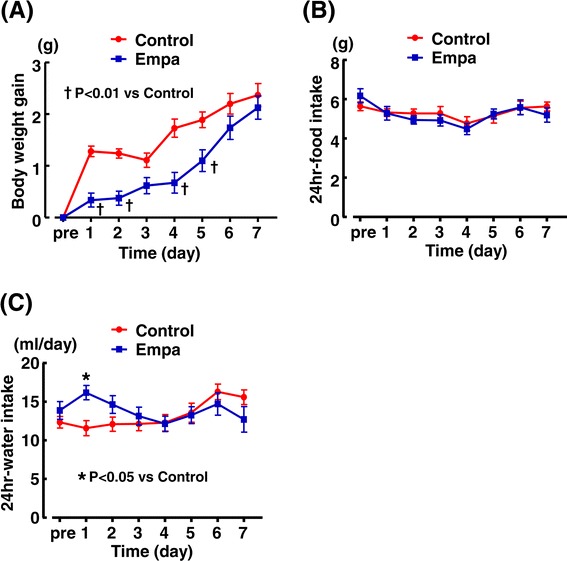
**Effects of short-term (7 days) empagliflozin administration on body weight gain (A), food intake (B) and water intake(C) of db/db mice.** Abbreviations used are the same as in Figure 2. *p < 0.05, †p < 0.01 vs control db/db mice. Values are mean ± SEM (n = 10-11).

As shown in Figure 3(A), body weight gain of empagliflozin group was less than that of control group on day 1-5 (except for day 3) after start of the treatment, but there was no significant difference in body weight gain between the groups on day 6 and 7. As shown in Figure 3(B), empagliflozin treatment did not affect food intake compared with control throughout the treatment. Water intake of db/db mice was significantly increased by empagliflozin only day 1 (Figure 3(C)).

**Figure 4:**
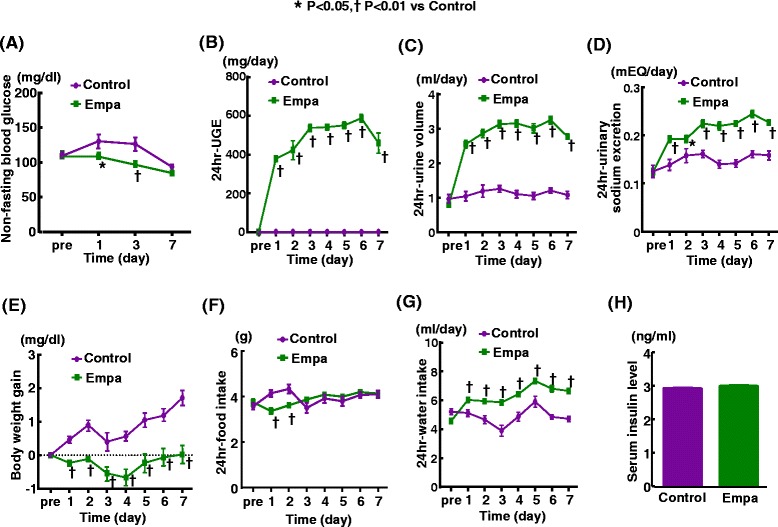
**Effects of short-term (7 days) empagliflozin administration on non-fasting blood glucose (A), 24 hr-urinary glucose excretion (B), 24 hr-urine volume (C), 24 hr-urinary sodium excretion (D), body weight gain (E), food intake (F), water intake (G), and serum insulin level (H) of db/m mice.** Abbreviations: db/m, non-diabetic db/m mice; Control, control (untreated) db/m mice; Empa, empagliflozin-treated db/m mice. *p < 0.05, †p < 0.01, vs control db/m mice. Values are mean ± SEM (n = 10).

We also examined the effect of short-term empagliflozin treatment in non-diabetic db/m mice. As shown in Figure 4, empagliflozin treatment significantly increased 24-hr urinary glucose excretion, urine volume, sodium excretion, and water intake, and significantly lessened body weight gain of db/m mice throughout the treatment. However, at the end of drug treatment (after 7 days of treatment), relative to control, empagliflozin did not significantly alter blood glucose levels (93.5 ± 3.2 mg/dl vs 84.7 ± 4.0 mg/dl) or serum insulin levels (2.92 ± 0.03 vs 2.99 ± 0.03 (ng/ml)) in db/m mice.

### The effects of long-term (10 weeks) empagliflozin treatment on hyperglycemia, serum insulin, body weight, urinary parameters, and blood pressure of db/db mice

**Figure 5:**
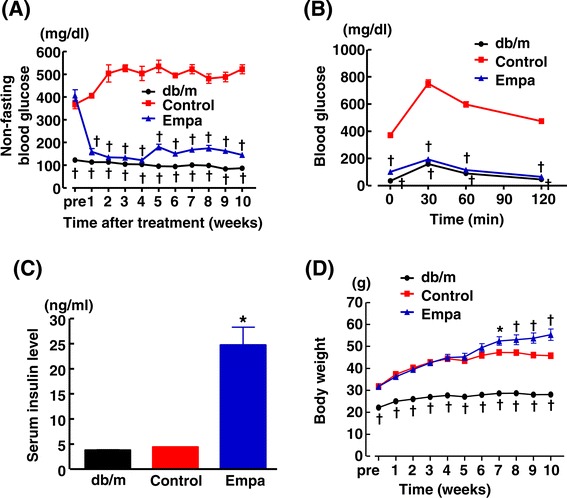
**Effects of long-term (10 weeks) empagliflozin administration on non-fasting blood glucose (A), oral glucose tolerance test (B), serum insulin levels (C), and body weight (D) of db/db mice.** Abbreviations: db/m, non-diabetic db/m mice; Control, control (untreated) db/db mice; Empa, empagliflozin-treated db/db mice. *p < 0.05, †p < 0.01, vs control db/db mice. Values are mean ± SEM (n = 9-11).

As shown in Figure 5(A), non-fasting blood glucose levels of db/db mice were much less in empagliflozin-treated group than in control group throughout 10 weeks of the treatment. Oral glucose tolerance test in Figure 5(B) indicates that empagliflozin improved glucose tolerance in db/db mice to almost similar levels to nondiabetic db/m mice. As shown in Figure 5(C), serum insulin levels were not different between db/m mice and control (untreated) db/db mice at the end of 10 weeks of the treatment (at the age of 17 weeks). However, serum insulin levels in empagliflozin-treated db/db mice were higher than those in control db/db mice (p < 0.05) (Figure 5(C)). Body weight of db/db mice subjected to empagliflozin treatment was significantly larger than those of control db/db mice after 7 weeks of the treatment (Figure 5(D)).

**Figure 6:**
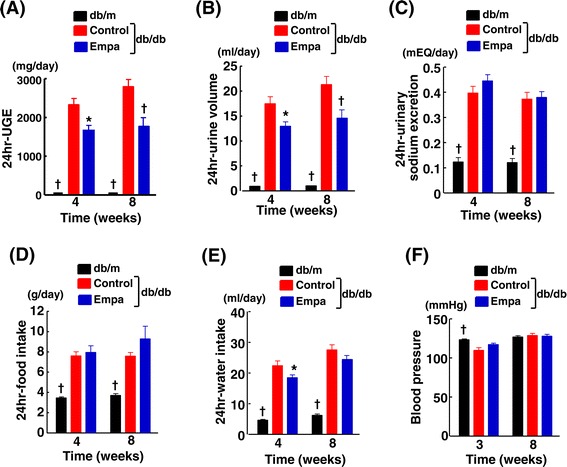
**Effects of long-term empagliflozin administration on urinary glucose excretion (UGE) (A), urine volume (B), urinary sodium excretion (C), food intake (D), water intake (E), and blood pressure (F) of db/db mice.** Abbreviations used are the same as in Figure 5. *p < 0.05, †p < 0.01 vs control db/db mice. Values are mean ± SEM (n = 9-11).

Figure 6(A)-(E) indicate the data obtained from 24-hour urine samples collected with metabolic cages at 4 and 8 weeks after start of empagliflozin treatment. Urinary glucose excretion and urine volume (Figure 6 (A) and (B), respectively) were significantly smaller in empagliflozin group than in control group. There was no significant difference in urinary sodium excretion (Figure 6 (C)), food intake (Figure 6 (D)) or water intake (Figure 6 (E)) between empagliflozin-treated and control db/db mice, except for the significant decrease in water intake in empagliflozin group at 4 weeks.

Figure 6(F) shows blood pressure at 3 and 8 weeks after start of the treatment. Blood pressure was comparable between control and empagliflozin-treated db/db mice.

### Effects of empagliflozin on cardiac injuries in db/db mice

**Figure 7:**
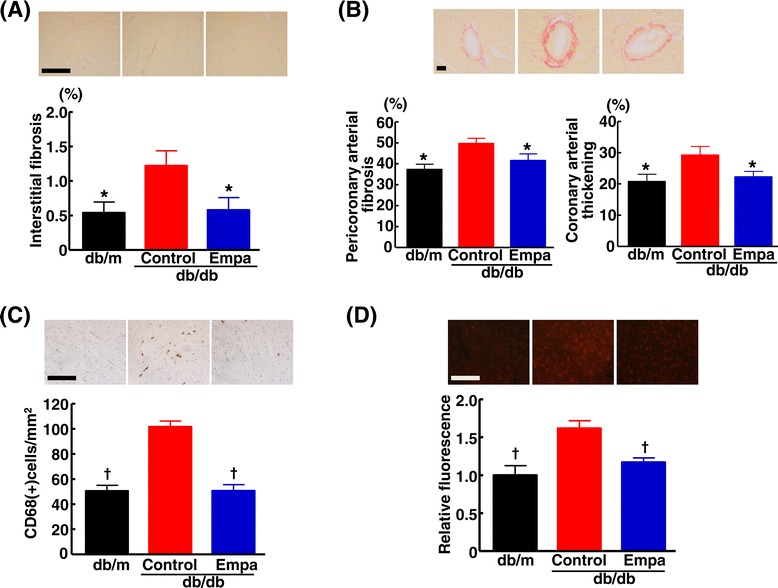
**Effect of long-term empagliflozin treatment on cardiac interstitial fibrosis (A), peri-coronary arterial fibrosis and coronary arterial thickening (B), cardiac macrophage infiltration (C) and cardiac superoxide (D) in db/db mice.** Upper panels in **(A)** and **(B)** indicate representative photomicrographs of cardiac sections stained with Sirius red. Upper panels in **(C)** and **(D)** indicate representative photomicrographs of cardiac sections stained with CD68 antibody and dihydroethidium, respectively. Abbreviations used are the same as in Figure 5. *p < 0.05, †p < 0.01 vs control db/db mice. Values are mean ± SEM (n = 9-11). Bar = 100 μm in **(A)**, **(C)**, and **(D)**. Bar = 50 μm in **(B)**.

Figure 7 indicates that cardiac interstitial fibrosis, peri-coronary arterial fibrosis, coronary arterial thickening, cardiac interstitial macrophage infiltration, and cardiac superoxide were significantly greater in db/db mice than in db/m mice. Ten weeks of empagliflozin treatment significantly ameliorated cardiac interstitial fibrosis (p < 0.05), peri-coronary arterial fibrosis (p < 0.05), coronary arterial thickening (P < 0.05), cardiac interstitial macrophage infiltration (p < 0.01), and cardiac superoxide levels (p < 0.01) in db/db mice.

### Effects of empagliflozin on vascular dilating function and aortic superoxide in db/db mice

**Figure 8:**
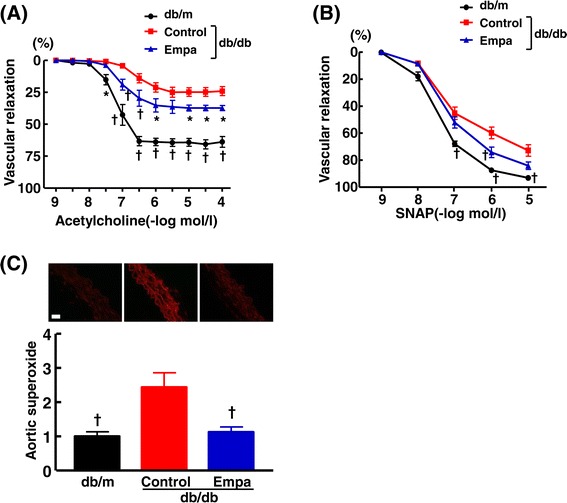
**Effect of long-term empagliflozin treatment on vascular relaxation by acetylcholine (A) and by SNAP (B), and vascular superoxide (C) in thoracic aortas of db/db mice.** Upper panels in **(C)** are representative photomicrographs of dihydroethidium-stained aortic section. Bar = 50 μm. Abbreviations used are the same as in Figure 5. *p < 0.05, †p < 0.01, vs control db/db mice. Values are mean ± SEM (n = 9-11).

As shown in Figure 8(A), vascular endothelium-dependent relaxation by acetylcholine in db/db mice was significantly impaired compared with nondiabetic db/m mice. Treatment with empagliflozin significantly ameliorated the impairment of vascular endothelial function compared with control db/db mice. Vascular endothelium-independent relaxation by S–nitroso–N-acetylpenicillamine (SNAP) was slightly but significantly impaired in db/db mice compared with db/m mice, and empagliflozin partially ameliorated the impairment of SNAP-induced vascular relaxation (Figure 8 (B)). As shown in Figure 8(C), aortic superoxide levels were significantly greater in db/db mice than db/m mice (p < 0.01). Empagliflozin significantly reduced aortic superoxide in db/db mice (p < 0.01).

### Effects of empagliflozin on cognitive function of db/db mice

**Figure 9:**
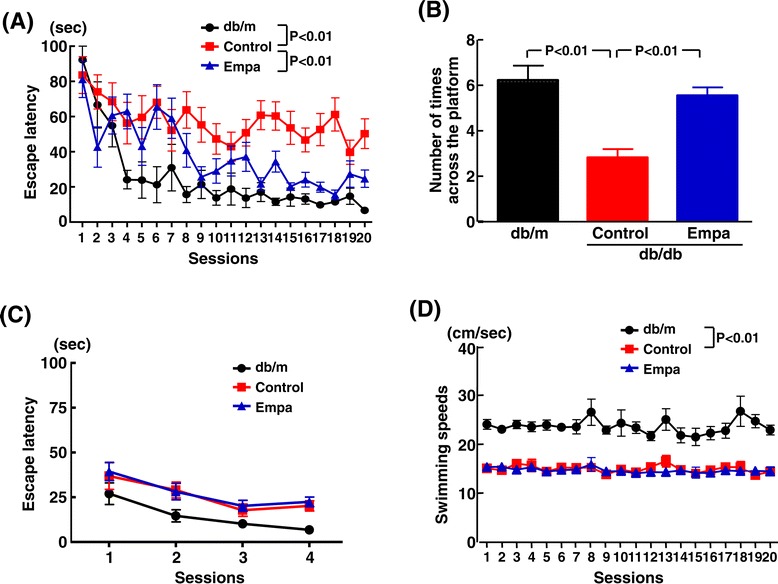
**Effect of long-term empagliflozin treatment on cognitive function of db/db mice estimated by Morris water maze test.** Cognitive function was evaluated by Morris water maze test at 9 weeks after the treatment. **(A)** indicates escape latency of the hidden platform test on 4 consecutive days (1-20 sessions). **(B)** indicates number of times across the platform in the probe test. **(C)** indicates escape latency in the visible test. **(D)** indicates swimming speeds. Abbreviations used are the same as in Figure 5. Values are mean ± SEM (n = 9-11).

As shown by the Morris water maze test in Figure 9, 16-week-old db/db mice showed significantly impaired learning and reference/working memory compared with the age-matched db/m mice. Escape latency of the hidden plat form test was greater in control db/db mice than in nondiabetic db/m mice (p < 0.01), and empagliflozin treatment significantly decreased the increased escape latency in db/db mice (p < 0.01) (Figure 9 (A)). Number of times across the platform in the probe test was smaller in control db/db mice than in db/m mice (p < 0.01), and empagliflozin reversed it in db/db mice (p < 0.01) (Figure 9 (B)). There was no difference between empagliflozin-treated and control db/db mice regarding escape latency in the visible test (Figure 9 (C)) or swimming speeds (Figure 9 (D)).

### Effects of empagliflozin on cerebral oxidative stress and brain-derived neurotrophic factor of db/db mice

**Figure 10:**
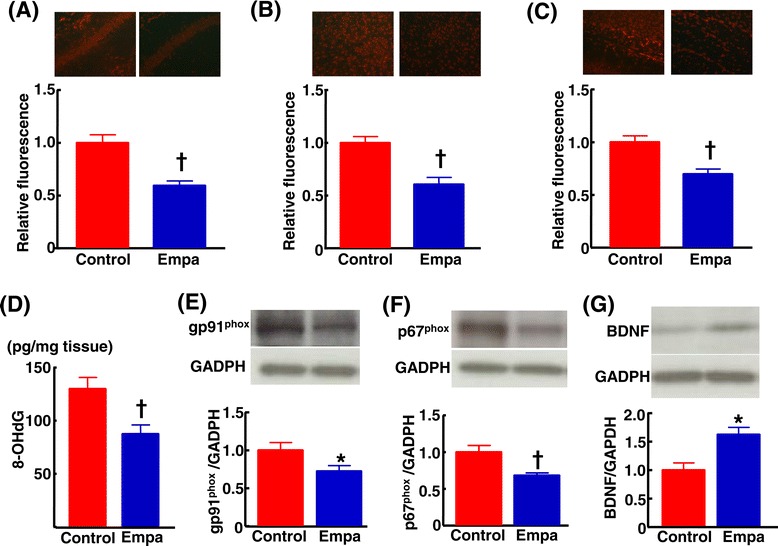
**Effect of empagliflozin treatment on cerebral superoxide ((A)-(C)), cerebral 8-hydroxy-deoxyguanosine (8-OHdG) (D), gp91phox (E), p67phox (F), and brain-derived neurotrophic factor (BDNF)(G) of db/db mice.** Upper panels in **(A)**, **(B)**, and **(C)** indicate representative photomicrograph of DHE-stained cerebral sections (hippocampus, cortex, and white matter, respectively). Upper panels in **(E)**, **(F)**, and **(G)** indicate representative Western blot band. Abbreviations used are the same as in Figure 1. Values are mean ± SEM (n = 11). *p < 0.05, †p < 0.01, vs control db/db mice.

Empagliflozin treatment significantly ameliorated cerebral superoxide (P < 0.01) (Figure 10 (A)-(C)) and 8-OHdG, a marker of DNA oxidative damage (P < 0.01) (Figure 10 (D)) in db/db mice. The attenuation of cerebral oxidative stress by empagliflozin was associated with the reduction of cerebral NADPH oxidase subunits, gp91 (P < 0.05) and p67 (P < 0.01) (Figure 10 (E)(F)). Moreover, empagliflozin treatment significantly augmented cerebral BDNF levels in db/db mice (P < 0.05).

### Effects empagliflozin on renal complication of db/db mice

**Figure 11:**
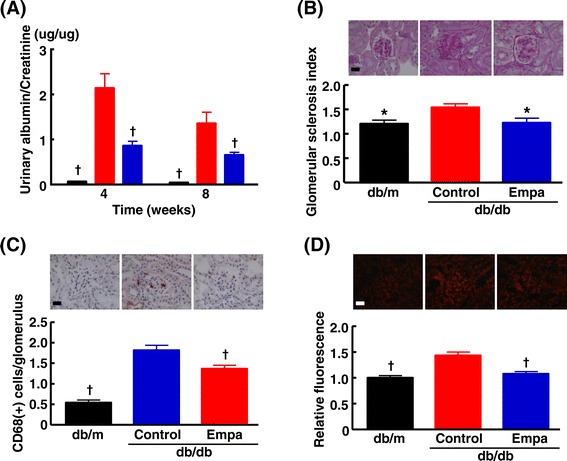
**Effects of empagliflozin on urinary albumin/creatinine ratio (A), glomerular sclerosis (B), glomerular macrophage infiltration (C) and glomerular superoxide (D) of db/db mice.** Urinary albumin/creatinine ratio in **(A)** was measured at 4 and 8 weeks after start of drug treatment. Upper panels in **(B)**, **(C)**, and **(D)** indicate representative photomicrographs of renal sections stained with PAS, CD68 antibody, and dihydroethidium, respectively. Abbreviations used are the same as in Figure 5. *p < 0.05, †p < 0.01 vs control db/db mice. Values are mean ± SEM (n = 9-11). Bar = 50 μm in **(B)**, **(C)**, and **(D)**.

db/db mice exhibited higher ratio of urinary albumin to creatinine excretions, higher glomerular sclerosis index, greater glomerular macrophage infiltration, and larger glomerular superoxide than nondiabetic db/m mice (Figure 11). Treatment of db/db mice with empagliflozin significantly ameliorated all these parameters related to glomerular injury.

## Discussion

Despite a number of therapeutic options, hyperglycemia in diabetic patients is often poorly controlled. SGLT2 inhibitors represent a novel class of drugs for treatment of type 2 diabetes and exert insulin-independent blood glucose lowering effects through the inhibition of glucose reabsorption and subsequent increase of urinary glucose excretion [12-14]. Furthermore, SGLT2 inhibitors do not increase the risk of hypoglycemia. Thus, SGLT2 inhibitors are expected to be a novel and promising agent for treatment of type 2 diabetes. Empagliflozin is a selective inhibitor of SGLT2 with good safety profile and does not require dose adjustments in patients with renal impairment [31-34]. The major findings of this study was that glycemic control with empagliflozin significantly ameliorated cardiac fibrosis and inflammation, coronary arterial remodeling, vascular dysfunction, and cognitive impairment in obese and type 2 diabetic mice. Furthermore, these beneficial effects of empagliflozin on cardiovascular injury and cognitive impairment were associated with the significant attenuation of oxidative stress in cardiovascular and cerebral tissues. These observations provide the experimental evidence supporting that empagliflozin may be a promising therapeutic agent for macrovascular complications and cognitive decline in type 2 diabetes.

Macrovascular disease is the leading cause of mortality in diabetic patients and the burden of cardiovascular disease due to diabetes mellitus has gone on increasing [1,2]. Although strict glycemic control is well established to reduce diabetic microvascular complications such as nephropathy or retinopathy, the benefit of glycemic control in preventing the macrovascular complications have been difficult to establish and there have been conflicting findings from previous large clinical trials [3-5,7]. Furthermore, dipeptidyl-peptidase IV inhibitors, a novel class of antidiabetic agent, have been recently reported to fail to improve cardiovascular outcomes in large clinical trials [6,8,12-14]. A multiple lines of experimental studies [17,20,21] on type 1 or type 2 diabetic animals show that glycemic control with SGLT2 inhibitors reduces the progression of diabetic nephropathy, one of the major microvascular complications attributable to diabetes. However, to our knowledge, it remains to be determined whether the SGLT2 inhibitor can protect against cardiovascular disease or cognitive impairment. Therefore, in the present study, we examined the effect of empagliflozin, a SGLT2 inhibitor, on cardiovascular injury and cognitive function in db/db mice, a useful model of obesity and type 2 diabetes.

In the present study, we obtained the evidence that empagliflozin ameliorated cardiac fibrosis and inflammation, coronary arterial remodeling, and the impairment of vascular dilating function in obese type 2 diabetic mice. Accumulating evidence [35-37] establish that hyperglycemia enhances tissue oxidative stress which plays a key role in the pathophysiology of diabetes mellitus and its complications. Therefore, in this study, we examined the effect of empagliflozin on cardiovascular oxidative stress in db/db mice, and found that empagliflozin attenuated the oxidative stress in cardiac and vascular tissues of db/db mice. We have previously reported that direct attenuation of oxidative stress in db/db mice with antioxidant causes the amelioration of cardiovascular injury, thereby demonstrating the direct involvement of oxidative stress in the progression of cardiovascular injury in db/db mice [28]. Taken together, our present observations support the notion that the amelioration of cardiovascular injury by empagliflozin might be attributable to the attenuation of tissue oxidative stress.

Diabetes is one of the major risk factors responsible for cognitive deficits such as Alzheimer’s disease and vascular dementia [9-11]. However, the potential benefit of anti-diabetic agents in prevention of cognitive deficits is unknown. Previous studies [38-40] demonstrate that db/db mice are characterized by impaired cognitive performance, thereby being regarded as a useful model to investigate cognitive decline attributable to diabetes. Therefore, in this study, we investigated the effect of empagliflozin on cognitive function in db/db mice. Of note, empagliflozin significantly prevented the progression of cognitive impairment (learning and reference/working memory) in diabetic mice, as shown by the findings on water maze test. To examine the potential mechanism underlying the amelioration of cognitive impairment by empagliflozin, we examined the effects of empagliflozin on cerebral oxidative stress. We found that empagliflozin treatment significantly attenuated cerebral oxidative stress and DNA oxidative damage in db/db mice, as shown by the reduction of cerebral superoxide and 8-OHdG, and this attenuation of cerebral oxidative stress was associated with the reduction of cerebral NADPH oxidase subunits, gp91phox and p67phox levels. Therefore, the improvement of cognitive function by empagliflozin seems to be attributed to the attenuation of oxidative stress. Moreover, we also examined the effect of empagliflozin treatment on cerebral BDNF, since BDNF [32], a key protein promoting memory and survival of neurons, is significantly reduced in diabetic patients and diabetic animals [31,41] including db/db mice [42] and the decrease in cerebral BDNF is shown to be associated with cognitive decline [41,43]. Interestingly, empagliflozin treatment significantly increased cerebral BDNF levels in db/db mice. These observations provided the evidence supporting that the increase in BDNF as well as the attenuation of oxidative stress appears to be responsible for the mechanism of prevention of cognitive impairment by empagliflozin. Thus, glycemic control with empagliflozin is proposed to be potentially a promising strategy for prevention of diabetes-induced cognitive deficits. However, future clinical study is needed to define our proposal.

In this study, we also examined the effects of empagliflozin on glomerular injury in db/db mice. We found that empagliflozin exerts protective effects against diabetic nephropathy, as shown by the reduction of urinary albumin excretion, glomerular sclerosis index, glomerular macrophage infiltration, and glomerular superoxide in db/db mice with empagliflozin. Our present results are in good agreement with previous reports regarding various SGLT2 inhibitors [17-19,41,44] including empagliflozin [16]. Thus, our present work confirms previous experimental findings supporting the benefit of SGLT2 inhibitors in prevention of diabetic nephropathy.

High blood pressure as well as diabetes is a major risk factor for cardiovascular disease and cognitive decline. Interestingly, clinical findings on type 2 diabetic patients demonstrate that SGLT2 inhibitors including empagliflozin significantly reduce blood pressure in diabetic patients [13,15]. Therefore, in this study, we also measured blood pressure in db/db mice. However, we found no significant alteration of blood pressure in db/db mice treated with empagliflozin. Thus, the protective effects of empagliflozin against cardiovascular and glomerular injuries, and cognitive impairment in db/db mice seems not to be attributed to blood pressure.

Accumulating clinical data show that SGLT2 inhibitors including empagliflozin reduces body weight in type 2 diabetic patients and glucose lowering action of SGLT2 inhibitors is insulin-independent [12-14,31]. In the long-term experiment of the present study, serum insulin levels were higher and the body weight was greater in empagliflozin-treated db/db mice than in control (untreated) db/db mice. However, it is unlikely that the higher insulin levels and greater body weight in empagliflozin-treated db/db mice is mediated by the direct action of empagliflozin. Along with aging, db/db mice are shown to exhibit the exhaustion of pancreatic β-cells because of long-standing glucose toxicity attributed to severe hyperglycemia and subsequently exhibit progressive decrease in serum insulin levels and loss of body weight gain [26,28,44]. Taken together with the findings that empagliflozin treatment substantially improved hyperglycemia, the greater body weight and the higher serum insulin levels in empagliflozin-treated db/db mice than in control (untreated) db/db mice can be explained by the significant prevention by empagliflozin of aging-associated decrease in serum insulin levels and of aging-associated loss of body weight gain in db/db mice.

The data on short-term (7 days) empagliflozin administration in db/db mice indicates that the significant increase in 24-hour urinary glucose excretion on day 1 after start of empagliflozin treatment was accompanied by not only the remarkable reduction of blood glucose but also the significant and transient increase in urine volume and urinary sodium excretion, and the significant decrease in body weight gain. However, from day 2 after the treatment, despite the remarkable continuous reduction of blood glucose, empagliflozin did not increase urinary glucose excretion, urine volume, and urinary sodium excretion in db/db mice, compared with control (untreated db/db mice). Furthermore, in the experiment on long-term empagliflozin treatment, urinary glucose excretion and urine volume were lower in empagliflozin group than in control group, although empagliflozin group had continuously much less blood glucose levels compared with control group. These paradoxical findings are in good agreement with previous report [44], and can be explained by the fact that the significant reduction of blood glucose levels attributed to the increased urinary glucose excretion with empagliflozin results in the significant decrease in the glucose content in glomerular filtrate and subsequently leads to the decrease in urinary glucose excretion.

## Conclusions

In conclusion, glycemic control with empagliflozin ameliorated cardiac fibrosis and inflammation, coronary arterial remodeling, vascular dysfunction, cognitive decline as well as glomerular injury in obese type 2 diabetic mice, thereby highlighting empagliflozin, a SGLT2 inhibitor, as potentially a promising agent for prevention of diabetic macrovascular disease and cognitive decline as well as nephropathy. However, no sufficiently powered clinical trial has yet elucidated the benefit of a SGLT2 inhibitor in prevention of diabetic macrovascular and microvascular complications, although large clinical trials to address this issue are now on going [45].

## Abbreviations

SGLT2: Sodium glucose co-transporter 2
UGE: Urinary glucose excretion
BP: Blood pressure
MWM: Morris water maze test
BDNF: Brain-derived neurotrophic factor
SNAP: S-nitroso-N-acetylpenicillamine
DHE: Dihydroethidium
PAS: Periodic acid-Schiff
